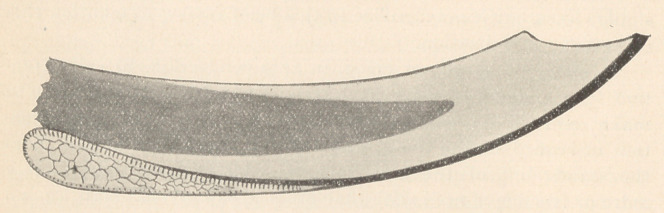# The New York Institute of Stomatology

**Published:** 1897-04

**Authors:** 


### Reports of Society Meetings.

### THE NEW YORK INSTITUTE OF STOMATOLOGY.

A REGULAR meeting of the Institute was held on Tuesday evening, January 5, 1897, at the office of Dr. George S. Allan, 51 West Thirty-seventh Street.

The President, Dr. George S. Allan, in the chair.

The minutes of the previous meeting were read and approved.

*Dr. W. St. George Elliott.*—I would like to exhibit a model which is going abroad this week, to be presented to the Odontological Society of London. It is a model of a case that I reported to this society several months ago, a young boy who was a patient in the Children's Hospital here, having a double ankylosis of the lower jaw. The case was operated on by Dr. Poor, of this city. I was present during the operation, in which the ramus was entirely detached on either side, making false joints. As can readily be imagined, I had great difficulty in taking an impression. I finally introduced a piece of shingle upon which was some impression material, and with this I succeeded in getting an impression. Kindly notice the remarkable shape of the jaws and the imperfect development of the lower jaw. The case has gone on successfully, and the young man has now very good use of his jaws.

*The President.*—Gentlemen, I take great pleasure in introducing to the members of the society and their guests our brother from Cambridge, Massachusetts, Dr. R. R. Andrews, who will now present us with a "Contribution to the Study of the Development of Enamel."

(For Dr. Andrews's paper, see page 205.)

*The President.*—This being a question of pure science, not necessarily being confined to dentists, your Executive Committee has invited Professor Wortman to open the discussion. Professor Wortman, although not compelled to earn his living by working upon the teeth, has devoted, perhaps, as much time and thought to the general subject of dental anatomy as any man in this country. He has studied the papers of Dr. Williams, as well as those of Dr. Andrews, and he is in a position to intelligently discuss the questions that have come up this evening.

*Dr. Wortman.*—Mr. President and gentlemen, I have listened with a great deal of interest to Dr. Andrews's paper and his demonstrations, and have also read with care the series of papers published in the *Dental Cosmos* by Dr. Williams. I regret to say that my personal experience of the points in controversy have not been sufficiently extensive to warrant me in speaking upon them with very much authority; at the same time there are some points that I may perhaps discuss.

Before taking up some of these points, it might be well for me to call attention to some of the great generalizations that have been made in the study of this subject, especially with reference to teeth as a whole. I will mention these generalizations, not in the order in which they have been made, but in the order of their importance.

That of the greatest importance, perhaps, was made by Professor Hertwig, in which he gave us a complete demonstration of a fact that a tooth originally pertains to the skin, that it is a dermal appendage, and that its connection with the jaw or with other parts of the skeleton of the mouth is merely a secondary affair.

The generalization next in importance was that made by Professor Huxley, in which he demonstrated the sources from which the different tissues of a tooth are derived. He it was who first gave us a clear and comprehensive understanding of the enamel organ in its relation to the production of enamel. He proved that its source is from epithelial or epidermal structures. He likewise demonstrated that the dentine organ is derived from the deeper-lying embryonic tissues of the jaw.

The next great generalization in the histology of the teeth was that of Tomes, in which he proved that enamel or an enamel organ is in some stage characteristic of all teeth. He established the fact beyond dispute that in all of those cases where the enamel is completely absent in the adult tooth, in its early stages of development, a rudimental enamel organ is present.

Coming now to the question of the structure of adult teeth, I will mention the great generalization made by Cope, in which he has demonstrated the fact from palæontological evidence that all complex tooth-structure has had its beginning in, and has been derived from, a simpler form, in many instances tracing it back to the stage of the simple cone. Although this idea is not fully accepted by all anatomists at the present time, yet it is such a strong hypothesis that it amounts to an almost complete demonstration.

Within the past five years we have had some remarkable additions to our knowledge of the embryology and succession of the teeth, made by certain German and English investigators. Among these I may mention Kükenthal, Röse, Leche, Tacker, Woodward, and others. These investigations go to prove that in all mammalia, instead of there being two sets of teeth, as we had heretofore commonly believed, there are really four, although two of them persist as mere vestiges. In the human mouth Röse claims to have found representatives of four sets of teeth in the early stages of development.

It has been often stated that there are cases in which a complete third succession is developed. If this be true, the explanation would not be difficult according to this view, for it would simply mean that one set of these vestigial teeth has been developed and calcified as ordinarily occurs with the permanent and milk set, with which we are already familiar. There are a number of other discoveries that have been made by these investigators along similar lines, but it must suffice in this brief review to mention this one great generalization.

It may not be out of place, in this connection, to call attention to a discovery that I have recently had the good fortune to make; and while it cannot be looked upon as a broad generalization in regard to the teeth, yet it is of considerable import from the stand-point of the morphologist. All present are probably more or less acquainted with that great group of mammals known as the "edentata." That character which distinguishes them most conspicuously is the entire absence of enamel upon any of the teeth. No clue to their ancestry has hitherto been discovered, and, if we can accept Tomes's law, they must have descended from ancestors with enamel-covered teeth. Indeed, Tomes found a vestigial enamel organ present in the early stages of development, and I can now demonstrate that their early ancestors had a fully developed enamel covering upon the teeth, quite like the ordinary mammal.

A few years ago it was believed and held to be a fact by nearly all dental anatomists that both the enamel and the dentine were formed by the progressive calcification of the dentine and the enamel organs. For my part, I regarded it as settled until Dr. Andrews established the fact beyond question, in regard to the enamel, that it is from materials secreted by the ameloblasts that the calcified product is formed. This discovery resulted in the complete overthrow of the old idea that the enamel cells themselves are calcified.

There are a number of matters of interest in Dr. Williams's papers, and, so far as I am able to judge, the fact of the greatest importance that he has shown is that to which Dr. Andrews has already called your attention,—viz , the nature of the stellate reticulum.

As regards these points in controversy between Dr. Williams and Dr. Andrews, I will address myself to only one or two. Let me first call attention to the question of the development of the enamel organ. Dr. Williams holds to the view, as I understand it, that blood-vessels are developed in this situation. This is a contradiction of all the teaching of histology up to the present time. He cites a number of sections taken from the enamel of the incisor of a rat to demonstrate his point. I have prepared and now exhibit a diagram of the longitudinal section of a persistently growing incisor of a rodent. In this diagram I wish to call attention especially to the position of the enamel organ, because in the formation of the enamel in a tooth of this kind, every stage is represented from the most embryonic condition to the completely formed tissue. It is a matter of great consequence, as I will attempt to show later, at what point the section is made, if we wish to understand clearly this question of development of blood-vessels in the stratum intermedium of the enamel organ.

But first let us ask ourselves, What is the enamel organ? If I were to attempt to define it, I would say that it is composed of those structures only which are derived from the original pouch or bud of epithelium which arises from the primitive fold. It is true that it may come to be altered later in the course of its development by the incorporation of other elements; but that by no means proves that blood-vessels are developed in this purely epithelial structure, as Dr. Williams would have us believe.

As long as the stellate reticulum is intact the ameloblasts are widely separated from the surrounding connective tissue; but as development proceeds the stellate reticulum disappears and the ameloblasts and stratum intermedium are brought in close contact with the connective tissue in which blood-vessels abound.

Now, as a matter of experience, I have found it difficult in this stage of enamel-formation to distinguish the boundary of the original enamel organ, and I suspect that the sections of the rodent incisor which Dr. Williams cites to establish his point are of this nature, and that the blood-vessels he finds have had their origin in the connective tissues.

We can probably also judge from this of the office of the stellate reticulum. It probably performs the functions of furnishing to the ameloblasts the necessary material for inaugurating the first steps in the formation of enamel, and its early disappearance would argue that its functions relate solely to the early embryonic stages.

I will invite attention next to the question of the proliferation or multiplication of the ameloblasts. In such a tooth as a persistently growing incisor of a rodent provision must be made for almost unlimited growth, seeing that the length of the tooth is many times greater during the course of the animal's life than it could possibly have been in the embryo. These appearances of bodies in connection with the nucleus described by Dr. Williams, and believed by him to be concerned in the production of enamel rods, I am inclined to think with Dr. Andrews represent but the process of cell multiplication.

Regarding the other points that have been raised in this discussion, I do not feel myself qualified to speak.

*The President.*—I now take pleasure in introducing our good friend Professor Peirce, of Philadelphia. I urged Professor Peirce very strongly to come to our meeting, knowing his great interest in these subjects, and he kindly consented.

*Dr. C. N. Peirce.*—I feel that the paper that has been read by Dr. Andrews, and the remarks of Dr. Wortman, have pretty well exhausted the subject, so far as any expressions that I might make in regard to the development of the tissue; but you will pardon a little inquiry in regard to one or two statements which seem to require some explanation.

As Dr. Wortman has said, in speaking of generalizations of the teeth, we have certain animals, such as the toad and dog-fish, where the teeth are simply specialized dermal appendages, and but little more than that; so, in the development of all teeth, we may call them specialized dermal cells, modified by location and function.

But the statement that struck me most forcibly to-night was that of Dr. Andrews regarding the formed enamel, which as soon as completed became non-living tissue,—that is, a deposition of lime in these columnar or specialized cells makes them non-living. I can hardly understand this statement, and am inclined to regard it a misnomer, because changes take place in the enamel all through early adult life, changes which are modifying it in color, density, and sensibility. Therefore, I should feel that the doctor hardly expressed what he meant when he spoke of the enamel as a non-living tissue. Dr. Alfred Gysi, of Switzerland, is making some very extended examinations in this direction, and some photographs which he sent this summer to Philadelphia show some protoplasmic masses. I should judge they were extending from the dentine up into the formed enamel. He contended that these masses were instrumental in carrying nourishment, or plasma containing lime salts, from the pulp in the dentine to the enamel. If this is the case, and I have no doubt of it, we have the tooth, after eruption, supplied and solidified until its induration is completed. I think every dentist recognizes the fact that as a patient advances in years the enamel grows more dense and more brittle, and contains less organic matter. That is certainly the experience of many with whom I have spoken. Another evidence of the vitality of the enamel is the fact that in persons of eighteen or twenty years of age we often see spots in the enamel, white or grayish in color, which in after years become yellow. I have always inferred that, as the plasma is the medium by which lime is carried from the blood into all these tissues, these were imperfectly calcified spots which subsequently become solidified, and in their solidification they became changed in color from white to yellow, because the secondary deposit was of that color. I think every observing dentist must recognize the fact that there is more or less change in the density and color of the enamel of the teeth as the individual advances in years. I have always looked upon the stratum intermedium as being but a progressive stage of development; that it is simply a modification of the stellate cells previous to their appropriation of the material to form the enamel. I have looked upon them, as Dr. Williams has said, as possessing the same function for enamel as the odontoblasts for dentine,—that is, a certain progressive stage of development instrumental in dentine-formation.

Another thing that I think has been clearly shown is the fallacy of the statement that the enamel rods are uniformly of hexagonal shape. We find them in all shapes,—six- as well as eight-sided. We must have such shapes as will fill in the spaces so that the tissue will be solid, because the enamel is the most dense structure in the animal economy.

*Dr. Andrews.*—Dr. Black is an investigator in this line. Has he noticed any great difference in the density of enamel of young and adult teeth?

*Dr. Peirce.*—Dr. Black has not noticed the difference that evidently exists and that should be recognized, nor does he see clearly regarding dentine, for he states that the density of the dentine is not materially increased. In this statement he entirely ignores the filling up of the tubules. Brittleness is certainly an indication of a less proportion of organic matter in the tissues. The books give three per cent. of organic matter in newly formed enamel, and twenty-eight per cent. in dentine. This as a person advances in years diminishes, though the percentage is necessarily small, and Dr. Black, with all his acuteness in investigation, may not be able to measure the change.

*The President.*—There was one point in this paper of Dr. Andrews which is of a purely histological character, and we have asked Dr. Graf, who is familiar with that line of research, to take part in the discussion.

*Dr. Graf.*—Mr. President and Gentlemen, it is a very hard matter for me to speak before this society, as I am entirely ignorant of dental anatomy, and I can therefore only discuss some points in the admirable paper of Dr. Andrews from a general cytological stand-point.

The first question raised is that of blood-vessels growing into an epithelial layer. The only similar case mentioned in biological literature was published by Ray Lankester, who believed to have found intraepithelial capillaries in the integument of the leeches. I have reason to believe that these capillaries are not situated between the epithelial cells, but directly underneath them in the connective tissue. The present case of capillaries in the stratum intermedium would therefore appear as a unique occurrence.

With regard to the origin of the calco-spherites, I should like to support the opinion of Dr. Andrews most emphatically that cell division or mitosis cannot have the slightest connection with this secretion.

Mitosis, or as we call it also karyokinesis, comprises a series of complicated processes which all tend towards dividing quantitatively and qualitatively the contents of the cell into two halves exactly alike.

The different phases of karyokinesis have been very clearly described by Dr. Andrews, and it is unnecessary to repeat his statement. One little point has since 1895 been worked out more carefully,—namely, the importance of the centrosome in cell division. This body seems to be a permanent specific organ of the cell, or we might call it a kynetic centre of the cell, and there are great doubts as to its nuclear origin during the prophase of division. It seems to be an independent formation.

During cell-division all the individual energies of the cell elements are set free and work in one direction, all tending towards mechanical distribution of the life elements or idiosomes in groups or systems of identical character. During this process we cannot suppose that secretion or other minor functions could go on as usual in the cell besides the cardinal function of mitosis.

I do not hesitate to say that mitosis, if such has ever been observed in the ameloblasts, which is exceedingly doubtful, cannot have the slightest connection with the secretion of calcareous matter from the cell.

It is, on the other hand, not improbable that the resting nucleus of the ameloblasts may have a great influence upon said secretion. It is well proved that the nucleus presides over the metabolic phenomena of the cells, whereas the cytoplasma is mostly the bearer of respiration and locomotion. Secretion and excretion are metabolic processes, and must be directly influenced by the nuclear substances. It is hard to say at the present time whether the excreted substances are formed within the nucleus or not, so much is sure that true chromatin is never used up in the formation of a secreted substance.

We find besides the chromatin another body in the nucleus,—the nucleolus. By a new method I have been able to show recently that the nucleolus consists in all the cases of a different substance than chromatin, which was proved by a sharply contrasting staining reaction of the two substances after a certain treatment.

I have found in cells, which I called reserve food-cells, that the nucleus undergoes great changes during the formation of the reserve food-granules. The chromatin granules melt together until the whole chromatin forms a homogeneous mass, whereas the nucleolus breaks up in a great number of minute granules which are dispersed throughout the whole cell. The cytoplasm of the cell is entirely used up in the formation of round, yellow globules, and it seemed to me as if the remains of the nucleoli were similarly used up in the formation of these food-globules.

I think we may have a similar case in the ameloblasts. By the aid of the cells of the stratum intermedium the lime salts may be brought in a structureless state into the ameloblasts, in which they are by the direct influence of the nuclear substances chemically transformed, and by subtile mechanical processes secreted in the form of globules,—calco-spherites.

Such cases are very commonly met with in secreting cells. There is another point which has not been quite clear to me,—namely, the net-work of fibres between which the calco-spherites are deposited. As far as I understood Dr. Andrews, he believes they are composed of protoplasma.

*Dr. Andrews.*—Formed from differentiated protoplasm.

*Dr. Graf.*—Dr. Andrews means they are exudates of protoplasm from the cells which become metamorphosed into some lifeless matter, something like a reticular formation, as in connective tissue.

*Dr. Andrews.*—It is the intercellular substance, formerly protoplasm, that is differentiated into fibres.

*Dr. Graf.*—Connective-tissue fibres may be understood to be transformed protoplasm, just as the contractile muscle fibrils are a metamorposed protoplasm.

*Dr. Andrews.*—Yes, as I understand it.

*Dr. Graf.*—If the connective-tissue fibres are metamorphosed protoplasm, then we have two kinds of fibres in the connective tissue,—extracellular fibres, which in this case would represent processes of the cells which have become transformed into a lifeless matter, and intracellular protoplasmic fibres, the regular cytoplasmic thread-work. These cytoplasmic threads form the ground substance of protoplasm, and the spaces between them are filled by a fluid, the cytolymph.

The cytoplasmic fibres may form an irregular net-work, or they may centre towards a central body, as in the astral figure, during mitosis, or they may even run parallel through the whole cell, as is the case in many secreting cells.

Such fibrous structures are also the ciliæ which protrude outside of the cell; but my view is that the ciliæ are lifeless, cuticular formations, and only in their basal pieces we have to see the moving, living part. I hold also that the extracellular fibres, as in the connective tissue and in Dr. Andrews's case, are cuticular formations, and not protoplasma.

*Dr. Andrews.*—I have been led to believe that they are formed from differentiated protoplasm, that they are lifeless, and become fully calcified.

*Dr. Graf.*—There is not the least doubt but that the fibres in question are lifeless, the only undecided point is whether they are lifeless from the beginning,—namely, that they are simply secreted by the protoplasm, or whether they were a first living exudate of protoplasm which became gradually transformed and calcified, and thus lifeless. This question is to my mind wholly undecided, and it is to be hoped that by new methods of investigation this point also might be elucidated.

The vice-president, Dr. E. A. Bogue takes the chair.

*Dr. George S. Allan.*—The two series of papers of Dr. Andrews and Dr. Williams cover the subject of the enamel and its development in a most beautiful and complete manner. I feel sure that if these two gentlemen, Dr. Andrews and Dr. Williams, could have studied this subject together, talking it over as they worked, there would not have been any great differences of opinion as to the points discussed to-night. They agree very closely in many matters, and I am convinced that their differences would have mostly disappeared had they been able to compare notes as they went along in their investigations.

Dr. Williams in his antagonism or dislike to the views of Professors Heitzmann, Abbot, and Bödecker is very positive in the expressions he makes use of; but I confess, for one, that I think Dr. Williams was justified to a great extent, for a more unwarranted endorsement of a book than that given by the National College of Dental Faculties to Dr. Bödecker's work I cannot conceive of, and that is Dr. Williams's excuse for his repeated references to Dr. Heitzmann's exploded reticulum theory. The investigations of the leading biologists and microscopists have shown that there is nothing in the theory of the least value. Dr. Andrews quietly ignores the subject of Professor Heitzmann's theory, and possibly that was the better way of expressing his own views.

The differences between Dr. Andrews and Dr. Williams seem to be largely in regard to the ameloblastic layer. Dr. Williams speaks of the ameloblastic membrane, and the thought comes up, if these are membranes what office do they perform? If the stratum intermedium secretes, through its cells possibly, some gelatinous fluid, how does that fluid pass through this membrane? It would be impossible, under the known laws of physics, for that fluid to pass through except on some principle of osmosis, and that would conflict strongly with Dr. Williams's conception of the duty of the stratum intermedium. The same difficulty confronts us when we consider the inner layer, or the inner membrane; that is, the membrane lying between the enamel cells and the forming enamel. Granting that that is a membrane, how can the forming material, which is secreted by the ameloblasts, pass through that membrane in any formed condition? It must act like a sieve, even if it be an open, fibrous structure; and if it is not an open, fibrous structure, and the fluid passes through by some principle of osmosis, then that completely destroys any possibility of the existence of a network of formed material that could construct a skeleton on which this beautiful structure is built. It is quite possible that the nucleus is in a large measure interested, if not directly certainly indirectly, in the production of the plasma that is to form the enamel prisms; but the theory of mitosis, as Dr. Andrews plainly shows, can have nothing to do with it. The nucleus most certainly is largely interested in the process of synthetic metabolism, more so than the cytoplasm of the cell, as recent investigations and discoveries plainly indicate, and so it would appear that Dr. Williams was not as careful as he ought to have been in seeking an explanation (foundation) for his views. Dr. Williams, I think, will acknowledge this when he reviews his work, and so no more need be said in reference to it.

While these photomicrographs of Dr. Williams, with some others that I have had presented to me by Dr. Mummery, of London, are the nearest to perfection of any that I have seen, I want to say most positively that the photomicrographs with which Dr. Williams illustrates his articles in the *Dental Cosmos* fall very far short of what he claims for them. I have looked them over very carefully, especially those of the formed enamel, and those which he claims show this fibrous matrix or skeleton net-work on which the enamel is said to be built, and either my eyes are very imperfect or the pictures do not show the net-work. I do not say that this beautiful structure does not exist, but so far as the photomicrographs are concerned they do not show it. Following his own line of study and his own illustrations, and what he has to say of them, I feel that it is doubtful whether there is any such beautiful structure. In a great many matters of this kind it is a question of interpretation. Dr. Williams may interpret a preparation one way and Dr. Andrews interpret it in another way; and in regard to this alleged skeleton net-work, I would protest against receiving it with full measure of authority.

One of my preparations of developing enamel shows the rods cut transversely and circular in form. They become hexagonal only when they lie close to one another, and compression has changed their shape. It is difficult to conceive how, at least in this preparation, a fibrous matrix could be developed. In the cut ends of the rods may be seen a dotted appearance as if some threads morphologically different from the rest of the prism were running through them. My study of this preparation and others inclines me to the belief that at this stage of development the rod is round, and no interprismatic threads can exist.

Before closing I want to show a series of photomicrographs which Dr. Mummery sent me a few years ago. They are equal to those of Professor Williams, and it is a pleasure for me to take this opportunity to show them in public, and at the same time to extend my thanks to Professor Mummery, which I have not had the opportunity of doing before in this public manner. Now these photomicrographs, which, as will be seen, are exceedingly beautiful, fall very far short of what may be viewed under a good compound microscope when the same tissues are examined, as any one having the slightest acquaintance with the use of the microscope will acknowledge.

My studies of the dental tissues have been mostly made with a P. & L. water immersion lens ($\frac{1}{8}''$), a most excellent glass, an apochromatic ($\frac{1}{12}''$) of same making, and a P. & L. achromatic condenser. I have never seen the superior to the last glass in clearness of definition.

*Dr. Watkins.*—Professor Wortman made the statement that there were in the human jaw the germs of four different sets of teeth, and that it was not an unusual thing for dentists to see the third set appear. I would like to ask Professor Wortman, or any other gentleman present, whether he has been led to believe that he has seen a third set of human teeth.

*Dr. Wortman.*—I have never seen a third set of human teeth, but we have the authority of Röse and others that there are four sets of germs present in the jaw in the early stage of embryonic growth. I depend upon their statements; and while I have never seen them myself, I am willing to accept their observations. I know that the statement has been frequently made, but I could not tell of any one who has ever seen a third set of teeth developed. I have heard it stated, and I believe it is held by some dentists, that a third set of teeth is not an impossibility.

*Dr. Andrews.*—I had a patient who had four molars on one side, and a more perfect set of all the teeth I never saw.

*Dr. Watkins.*—I have seen what appeared to be four lateral incisors in the upper jaw, and all apparently perfect. That is the nearest I have ever got to the third set of teeth.

*The President.*—We will now ask Dr. Andrews to say what he desires in closing.

*Dr. Andrews.*—Mr. President, I have little more to say. In the discussion the various points have been well covered. In regard to what Professor Peirce has said about the persistency of the organic matter in the enamel, I may call his attention to Dr. Black's recent investigations. He has found that there is very little difference between enamel of soft and enamel of hard structure. His experiments proving this were made with the finest modern appliances, and were thoroughly scientific.

Charles S. Tomes, of London, after recent analysis, endorses this. He says enamel is practically an inorganic tissue, there is not enough organic matter in it for quantitative estimation. He gives us clearly to understand that that which has generally been figured as organic matter is really water in combination with calcic matter. This statement of Mr. Tomes was made before the Odontological Society of London in 1896.

There are undoubtedly some points concerning the formation of enamel that we do not clearly understand, at least I do not. We find organic bodies running into the substance of the enamel from the dentine, but this is not an enamel substance, it comes from the dentine before the parts calcified. It is a subject that needs further investigation. I believe with Professor W. X. Sudduth, that the enamel is merely a coat of mail to protect the dentine, and with Tomes, Black, and Williams, that enamel is practically an inorganic substance.

The question asked by Dr. Allan, as to the chemical nature of these globular bodies, would require a great amount of work to answer intelligently, work of a somewhat different kind from that which I have been doing. It would require the services of the histo-chemist. When the chemistry and histology of the tissues are studied together, we shall arrive at more exact conclusions. I have not the ability or the time to go into such a series of investigations. I do not recall any other point I wish to speak about.

*Dr. Charles F. Allan.*—Mr. President, the paper we have just listened to by Professor Andrews is a very valuable one, and it has also been interesting almost beyond precedent because of the beautiful way it has been pictorially illumined. The labor necessary to prepare such a paper is simply immense. Only the labor of love could have produced it, a labor that knows no fatigue and never wearies.

I know I voice the sentiment of all present when I move a vote of thanks to Dr. Andrews for his extremely instructive paper; and I would also include Professors Wortman and Peirce and Dr. Graf, who have so kindly and helpfully participated in the discussion.

The motion was carried unanimously.

Adjourned.

S. E. DAVENPORT, D.D.S., M.D.S.,
*Editor The New York Institute of Stomatology.*

### ANNUAL DINNER—HONORS TO DR. BENJAMIN LORD.

NEW YORK, February 23, 1897.

ON the evening of February 12, the newly appointed holiday to commemorate the birthday of Abraham Lincoln, The New York Institute of Stomatology held its first annual dinner.

The incentive to this dinner was not exactly what the cynic described as the incentive to matrimony,—"an insane desire on the part of a young man to pay a young woman's board,"—but it was a similar insane desire on the part of all the members to present to Dr. Benjamin Lord, the retiring president, a certificate of good character (as though he needed it), enfolding within every signature a certificate of affection.

The dinner was intended as a family affair, so quarters were selected that did not admit of many invitations outside of the membership, and, as usual with the Institute, there was no special effort and no flourish of trumpets at all, but there sat down to the prettily decorated tables at the Knickerbocker Athletic Club thirty-five gentlemen to do honor to the occasion.

Dr. George S. Allan, this year's president of the Institute, presided, the honored guest, our ex-president, Dr. Lord, sat at his right, and Dr. Eugene H. Smith, dean of the Dental Department of Harvard University, sat at his left.

Facing them, at the foot of the table, which was of a T-shape, sat Dr. C. A. Woodward, chairman of the Executive Committee and of the special committee for the dinner.

It is needless to commend the dinner itself, "actions speak louder than words," and the generally contented hum of conversation showed the satisfaction of the diners.

When coffee was reached, the president called upon Dr. S. E. Davenport, the editor of the Institute and chairman of the committee to procure the testimonial, to take the floor.

Dr. Davenport, in a few well-chosen sentences, recited some of the difficulties that had been encountered in the organization of this new society and the manner in which our venerated ex-president had met and conquered them.

He brought to the minds of the gentlemen present the continuous labors of Dr. Lord in the interests of the Institute, and shortly closed by offering to him an engrossed testimonial, appropriately worded and prettily framed, and signed by every active member of the Institute.

Dr. Lord, in replying to this very delicate and touching tribute, was not at all so sure that he had always been good, and was quite inclined to depreciate the merits of his own administration, something to which the members were not inclined to listen. Dr. Lord presently took refuge in the expedient of passing to Dr. Elliott, the secretary, an unpublished history of certain lines of American dentistry of great interest, enumerating among other things the fact that Longfellow's hero, Paul Revere, was a dentist, and giving one of Revere's advertisements taken from the *Boston Gazette* of December 19, 1768. This history also set forth in some detail some of the difficulties experienced by the Father of his Country when once he tried to change dentists and have a new set of teeth made, and further explaining how the Stuart portraits of Washington obtained the horrible mouth which characterizes them, by means of cotton rolls which had been placed beneath the lips in the vain effort to restore a former expression.

After this history, Dr. Smith, of the Harvard School, made a short address of congratulation to the Institute upon the progress it had made in assuming broader and higher grounds of study than are usually found among dental bodies, and instanced the present company assembled in proof of their success.

He then presented for the examination of the Institute some plaster models of a case of irregular teeth where one or two teeth were missing.

With these models he presented four X-ray photographs of the same mouth, taken by our colaborer, Dr. Clapp, of Boston, and exhibiting the missing teeth well up in the alveolus, thus giving some indications as to the mode of treatment that may be possible.

Dr. Farrar was then called upon and gave an interesting account of dental historical matters which were quite new to most, if not all, of those present.

It seems that Dr. Farrar went quite deeply into dental history at one time, but eventually turned his attention more particularly to dental orthopædics, of which his great work is the result.

Dr. Cook, of Brooklyn, was called on for a few words, but thought the occasion too happy to be added to by any of his immature remarks, and no persuasion or cheering could make him think to the contrary.

The president thereupon began to abuse one of the honorary members, who in pure self-defence had to rise for reply, when Dr. Allan introduced Dr. Dawbarn, professor of Surgery at the Polyclinic College and Hospital. The doctor spoke but a very short time, keeping his auditors wide awake and sharply attentive, and at an early hour, as becomes the young, this youngest of the dental societies went home.

## Figures and Tables

**Figure f1:**